# Bioactive Compounds in Plasma as a Function of Sex and Sweetener Resulting from a Maqui-Lemon Beverage Consumption Using Statistical and Machine Learning Techniques

**DOI:** 10.3390/ijms24032140

**Published:** 2023-01-21

**Authors:** Diego Hernández-Prieto, Pablo S. Fernández, Vicente Agulló, Cristina García-Viguera, Jose A. Egea

**Affiliations:** 1 Phytochemistry and Healthy Food Lab (LabFAS), Department of Food Science and Technology (CEBAS-CSIC), Campus Universitario Espinardo-25, 30100 Murcia, Spain; 2 Agronomic Engineering Department, Universidad Politécnica de Cartagena (UPCT), Paseo Alfonso XIII, 48, 30203 Cartagena, Spain; 3 Associated Unit of R&D and Innovation CEBAS-CSIC+UPCT on “Quality and Risk Assessment of Foods”, CEBAS-CSIC, Campus Espinardo-25, 30100 Murcia, Spain; 4 Human Nutrition Unit, Department of Food & Drug, University of Parma, 43125 Parma, Italy; 5 Fruit Breeding Group, Department of Plant Breeding, CEBAS-CSIC, University Campus of Espinardo-25, 30100 Murcia, Spain

**Keywords:** dietary intake, anthocyanins, flavanones, biostatistics, machine learning, clustering, feature selection, data imputation

## Abstract

The present study analyses the effect of a beverage composed of citrus and maqui (*Aristotelia chilensis*) with different sweeteners on male and female consumers. Beverages were designed and tested (140 volunteers) as a source of polyphenols, in a previous work. Plasma samples were taken before and after two months of daily intake. Samples were measured for bioactive-compound levels with metabolomics techniques, and the resulting data were analysed with advanced versions of ANOVA and clustering analysis, to describe the effects of sex and sweetener factors on bioactive compounds. To improve the results, machine learning techniques were applied to perform feature selection and data imputation. The results reflect a series of compounds which are more regulated for men, such as caffeic acid or 3,4-dihydroxyphenylacetic acid, and for women, trans ferulic acid (TFA) or naringenin glucuronide. Regulations are also observed with sweeteners, such as TFA with stevia in women, or vanillic acid with sucrose in men. These results show that there is a differential regulation of these two families of polyphenols by sex, and that this is influenced by sweeteners.

## 1. Introduction

Nowadays, the dietary habits of Western society include a wide range of plant-based beverages rich in bioactive compounds such as (poly)phenols. These compounds are beneficial to human health, mainly due to their antioxidant activity [1,2]. In contrast, the significant presence and consumption of soft drinks with high content of sweeteners and other additives may present an issue regarding important challenges in public health at present, e.g., overweight, cardiovascular problems, diabetes mellitus, etc. Therefore, promoting the consumption of plant-based beverages, gaining a better understanding of their characteristics and thereby making the potential product as healthy as possible, is a research objective. Likewise, it is important to provide insights into alternative sweeteners to sucrose, since sweeteners may affect the bioavailability of (poly)phenols present in beverages, as suggested by Agulló et al. [3], and can also make the beverages more palatable to consumers.

In addition, nutrition-intervention studies usually do not consider differences concerning the sex of the consumer. Even if there is parity among volunteers, the results are analysed as a whole, losing sight of the more-than-possible differences between men and women. Traditionally, diets, the posology of drugs, etc. are men-oriented, [4] falling into a common error called gender blindness. Hence, studies are only conducted on menopause, pregnancy, or certain types of cancer that only affect the female anatomy. For nutrition, this lack of sex perspective is even more pronounced [5]. In this sense, physiological or hormonal differences between the sexes can influence the assimilation of bioactive compounds [6]. 

In this respect, the present work intends to estimate the potential difference in bioactive-compound absorption between the sexes, after consumption of a maqui-citrus drink sweetened with stevia, sucralose or sucrose, previously designed in our laboratory. The data analysed comes from a two-month longitudinal intervention-study carried out by our group, where 140 overweight volunteers ingested the different drinks, one per day, and plasma was collected at the beginning and end of the trial [3,7]. This study highlighted maqui and citrus as relevant sources of bioactive and bioavailable (poly)phenols, mainly anthocyanins and flavanones, respectively, and proposed stevia and sucralose as alternative sweeteners, as drinks elaborated with them showed a higher bioavailability of phenolic compounds than sucrose-sweetened drinks. In [8], a positive relationship between glycaemic response and stevia was assessed, but it was not enough to link the sweetener with determinate diseases. However, in these studies with the maqui citrus beverage, the results were analysed with basic statistical methods that did not allow for the assessing of clear differences between the sexes. 

Consequently, the present work aims to apply a new, more complex set of statistical tools to the previous results, to identify potential sex differences in the metabolism of phenolic compounds. From here on, the issues to be addressed are (i) the difference in metabolites between the sexes, dependent and independent of sweetener, (ii) the potential relations between metabolites, and (iii) the effect of the sweetener on the metabolites, regardless of sex. Issues (ii) and (iii) were explored in previous work related to this trial [3], but their insights can become more detailed with statistical and machine-learning techniques applied to the data. Finally, issue (i) is important for completing this type of experiment by offering a new perspective that adds information to the results and enables the potential applications of this beverage and the results of its effects on the metabolism of the different sexes.

## 2. Results

### 2.1. ANOVA Results

#### 2.1.1. Anthocyanin Set

**Figure 1:**
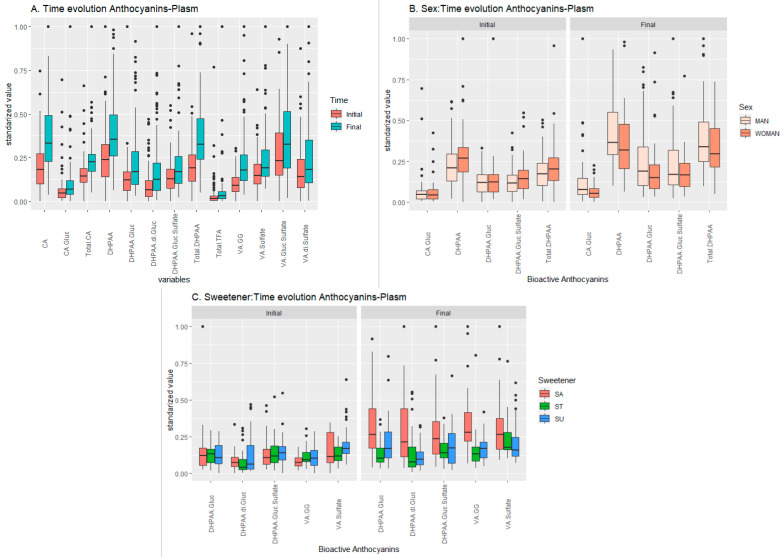
Temporal evolution of the effect of the beverage over the response (metabolic values) in the anthocyanin-plasma set. (**A**) Effect of the beverage over time, (**B**) effect of the interaction sex–time, and (**C**) effect of the interaction sweetener–time.

The anthocyanin content of the beverages was characterised by individual anthocyanins, in the following decreasing-concentration order: delphinidin 3,5-*O*-di-glucoside > delphinidin 3-*O*-sambubioside-5-*O*-glucoside ≈ delphinidin 3-*O*-glucoside > co-eluting cyadinin 3-*O*-sambubioside-5-*O*-glucosidec and cyanidin 3,5-*O*-di-glucoside > delphinidin 3-*O*-sambubioside > cyanidin 3-*O*-glucoside > cyanidin 3-*O*-sambubioside [3]. Analysing the anthocyanin dataset, corresponding to the concentration of the anthocyanin metabolites, it was observed that almost all their metabolites (the exceptions were trans-ferulic acid- glucuronide (TFA-G) and TFA-sulfate (TFA-S)) with available data were positively influenced by time (Table 1 and Figure 1A). The strongest interactions measured were 3,4-dihydroxyfenylacetic (DHPAA), total DHPAA and vanillic acid di-glucuronide (VA-GG), with the concentration of these metabolites being much higher at the end than at the start time. 

Moreover, CA-glucuronide (CA-G), DHPAA, DHPAA-GS and total DHPAA were influenced by sex according to ANOVA, as an increase in the concentration of these metabolites within men but not in women was observed (Figure 1B). 

Last, the concentrations of DHPAA-G, DHPAA-GG, DHPAA-GS, VA-GG and VA-sulfate (VA-S) were increased by sweetener–time interaction, mainly when sucralose was added (Figure 1C), according to ANOVA. On the other hand, the results from the pairwise *t*-test for this dataset (Table 1) confirmed the influence of the beverage (represented as the time factor), regardless of sweetener, over almost all the compounds studied. Moreover, in agreement with ANOVA results, their concentration increased over time in sucralose-added beverages. 

Regarding sex–time interaction, males were mainly influenced, except for total DHPAA, where concentration was increased in both males and females. This is a surprising result, since sex was selected as a differential factor to influence the concentration of total DHPAA (the answer may rely on the order of the *p*-value: 10^−4^ for females and 10^−11^ for males). Sucrose seems to be the most important sweetener regulator in the anthocyanin metabolites present in plasma, while stevia played a lesser role and sucralose played an even smaller one. The three-way interaction appears more often in the male-sucrose case, which means that males who consumed the beverage sweetened with sucrose raised their levels of CA, total CA, DHPAA-G, DHPAA-GG, DHPAA-GS, DHPAA-SS, total DHPAA, VA-GG and VA-GS. Interestingly, the effect in women of the beverage with stevia added over the aggregate of TFA and its derivatives (total TFA) was quite relevant, reaching a 10^−4^ order in its *p*-value (*p*-values for *t*-test are detailed in Supplementary Material, Table S1).

#### 2.1.2. Flavanones Set

**Figure 2:**
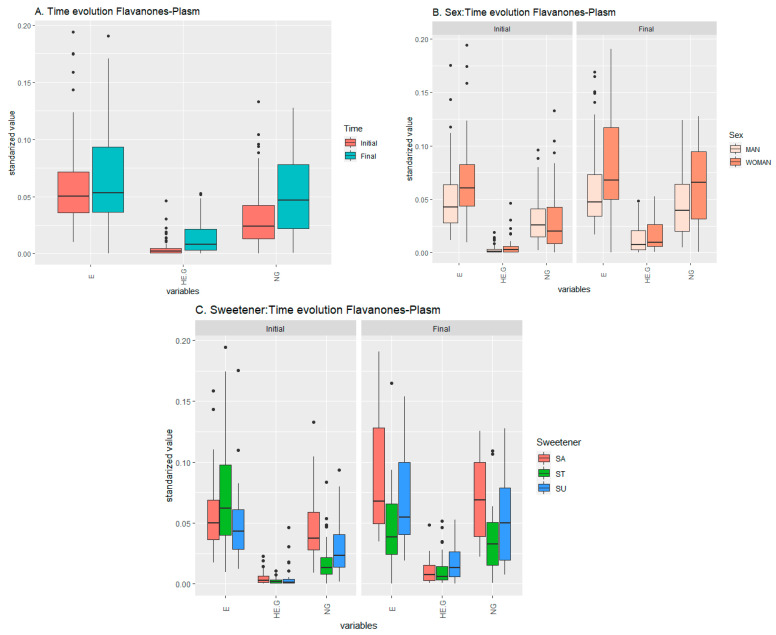
Temporal evolution of the effects of the beverage over the response (metabolic values) in the flavanones plasma set. (**A**) Effect of the beverage over time, (**B**) effect of the interaction sex–time, (**C**) effect of the interaction sweetener–time.

With respect to flavanones, the individual compounds were found, in the following decreasing-concentration order: hesperidin (hesperetin 7-*O*-rutinoside) > eriocitrin (eriodictyol 7-*O*-rutinoside) > narirutin (naringenin 7-*O*-rutinoside) > *O*-tri-glycosyl-naringenin [3]. Looking at the flavanones dataset, corresponding to the concentration of the metabolites, the analysis of variance only described as relevant the time factor, i.e., the consumption of the beverage regardless of the sex of the consumer or the sweetener added. This was the case just for eriodyctiol (E) and naringenin glucoside (N-G) (Table 2), while homoeriodyctiol glucuronide (HE-G) seemed to increase its level as a function of time, and the *p*-value for time factor in HE-G (0.115) was higher than 0.05 (Figure 2A). The results extracted from the pairwise *t*-test strengthen the ANOVA results for the time factor, which means that the concentration of these metabolites is increased at the final time. Those results also revealed a potential positive influence in E for males and for females in N-G and HE-G (Figure 2B), which was not endorsed by ANOVA. They also pointed out the effects of sucralose and stevia over the compounds’ concentrations as more relevant than those of sucrose (Figure 2C). There was almost no interaction between specific sweeteners and the different sexes. Although, as mentioned above, the *p*-value of HE-G was not less than 0.05 in the ANOVA results, it was included in the boxplots because its results in the *t*-tests were quite relevant (Supplementary Material, Table S2).

### 2.2. Clustering Results 

Clustering techniques group individuals based on different measures of distance between the values of the features of individuals. Through them, associations between compounds were obtained at different times, and it is possible to observe how individuals were grouped throughout the experiment. In this work, the graphical representation for clustering was a principal-component-analysis projection representing the individuals with arrows representing the bioactive compound with its importance in the groups. Ther clustering technique chosen and the number of clusters are reported in Table 3.

#### 2.2.1. Feature Selection

The results of the selection for anthocyanin data are reported in Table 4. For both sample times, TFA-S and total TFA were maintained, which may indicate the importance of these values in explaining the biological variability of both the sex at the initial time and the effect of the sweetener on the different sexes at the final time. 

#### 2.2.2. Anthocyanins Set

**Figure 3:**
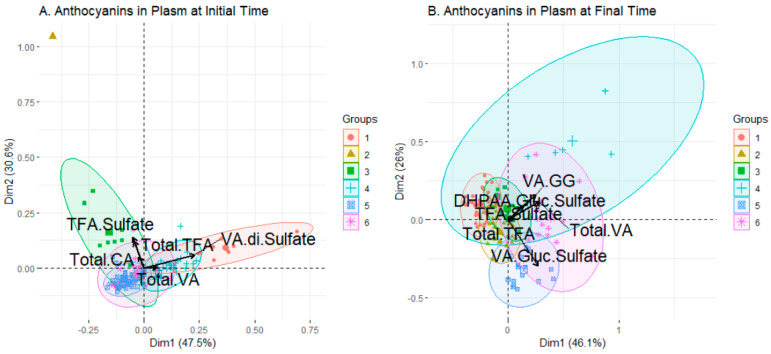
Biplot of the groups generated by clustering analysis applied to anthocyanin data set before (**A**) and after (**B**) consumption of the beverage. In black, the arrows showing the contribution to the composition of the clusters of the variables.

**Figure 4:**
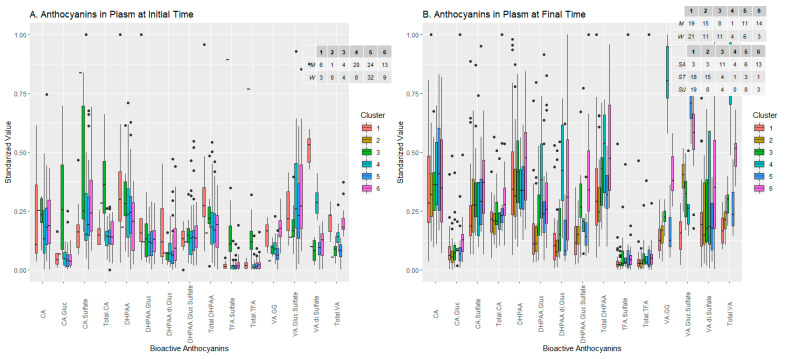
Levels of bioactive anthocyanin molecules within the groups generated by clustering analysis before (**A**) and after (**B**) beverage consumption, and composition of groups in terms of the sexes and sweeteners added to the drink.

In the anthocyanin set, with more existing variables, we expected to find distributions with more noise and which were less defined, which is why feature selection was applied. At the initial time, six clusters were defined (Figure 3A): two which were more dispersed, with fewer individuals (clusters 1 and 3), three with more points and which were partially overlapping (clusters 4, 5 and 6) and one cluster of a single individual (observation 81, cluster 2), far away in the PCA projection from the rest of the clusters. On the other hand, the variables TFA-S, total TFA and total CA defined clusters 2, 3 and partially 5 and 6, while the variables total VA and VA-SS defined clusters 1 and 4 in a more defined way. Observing the anthocyanin levels in each cluster (Figure 4A) confirmed what was represented in the biplot, i.e., that clusters 2 and 3 had a strong relationship with TFA-S, total TFA and total CA, while for clusters 1 and 4 the relationship was mostly with VA-SS, with total VA also important in cluster 6. The last three clusters included most of the population studied, with clusters 4 and 6 formed mostly by men and cluster 5 mostly by women. The latter did not have any composite whose value stood out above the rest, which may support the ANOVA results in which the women had no particular influence over any concentration of compound, and it was mainly the men who presented a strong differential influence on the levels of concentration of the listed compounds, as seen in clusters 4 and 6.

For the final time, where sweeteners already influenced the arrangement of the clusters in the PCA projection (Figure 3B), a group was observed with some highly dispersed individuals (cluster 4), two more intermediate in both individuals and dispersion (clusters 5 and 6) and three groups with many highly concentrated individuals with partial overexposure (clusters 1, 2 and 3). Cluster 5 was strongly defined by VA-GS, which also influences cluster 2, to a lesser extent. On the other hand, DHPAA-GS, total VA and VA-GG determined clusters 4 and 6, in addition to 1 and 3, to a lesser extent. The arrows of TFA-S and total TFA indicate little intensity in the composition of the clusters, which is striking regarding the compounds selected for both conditions. This seems to indicate that the rest of the compounds have become more important, due to the beverage-ingestion process. Looking at the compound levels present in the clusters (Figure 4B), the very low levels of TFA-S and total TFA are striking, with no cluster standing out from the start. The number of individuals in the clusters is quite balanced (except for cluster 4). In the composition of the sexes and sweeteners it can be seen that clusters 3 and 4, which have a higher presence of females and sucrose-consumers, have even total DHPAA levels, and whenever cluster 4 shoots up by one value (as happens in DHPAA-GS, VA-GG and total VA), cluster 3 stands out as behind the rest of the groups, although cluster 6, which contains higher numbers of males but an equal majority of sucrose, always tends to be between these two. This may indicate that the influence of sucrose on these compounds is strong, regardless of sex. Clusters 2, 5 and 6, with most males but different sweeteners, share a high value of VA-GS, which may indicate that the regulation of this compound is due more to sex than to the sweetener. 

Analysing observation 81, which covers all of cluster 2 at the initial time, very high levels of TFA-S and total TFA were observed, which corresponds to the values of the arrows reflected in the biplot. At the final time, this observation belonged to cluster 1, which was very concentrated around the centre, indicating that it presented more average values, and this was seen in the values of observation 81, which, in the case of TFA-S and total TFA, were almost half the values at the initial time. The corresponding subject is a male, who ingested the sucralose-sweetened beverage, and whose anthocyanin levels tended to increase with beverage consumption except for the set of TFA-G, TFA-S and total TFA. This may indicate metabolic oddity, experimental error, or up-regulation of TFA by sucralose. 

#### 2.2.3. Flavanones Set

**Figure 5:**
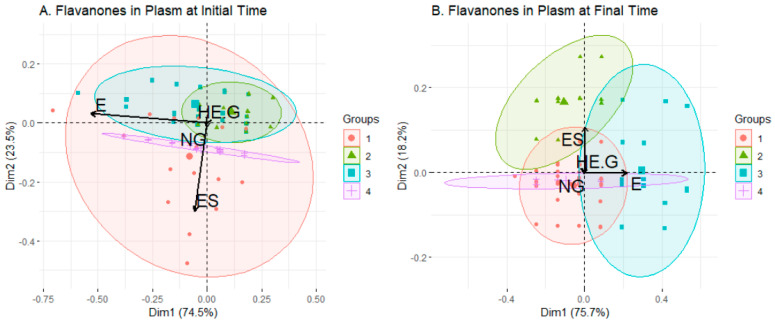
Biplot of the groups generated by clustering analysis applied to flavanones data set before (**A**) and after (**B**) consumption of the beverage. In black, the arrows showing the contribution to the composition of the clusters of the variables.

Only two variables seemed to be relevant in terms of the composition of clusters in both sampling times: E and ES, which match with the results of the Boruta algorithm (see Supplementary Materials, Table S1). Following this explanation, in the biplot referent for initial time (Figure 5A), one dispersed and less-crowded cluster (cluster 1), which in its range covers the rest of the clusters, had its composition influenced by both relevant variables. Compound E determined the composition of clusters 3 and 4, the latter having a rather peculiar shape, as discussed below. Lastly, cluster 2 was completely included in cluster 3, which in turn was encompassed in cluster 1, and it was not well defined by any compound. The clusters were quite balanced in the number of individuals, with clusters 1 and 4 slightly smaller than 2 and 3. Composition regarding sex was also balanced, and the major difference was found in cluster 4, with 14/9 in males/females. The levels of flavanones present in the clusters reflected the information covered in the biplot, with cluster 1 showing high levels of both compounds E and ES, clusters 2 and 4 with the same level of E, and cluster 3 not well determined by any flavanone. Cluster 4 had a remarkably low dispersion in the boxplot for ES, while the range of E was wider, which probably explains the almost linear shape of the distribution. 

**Figure 6:**
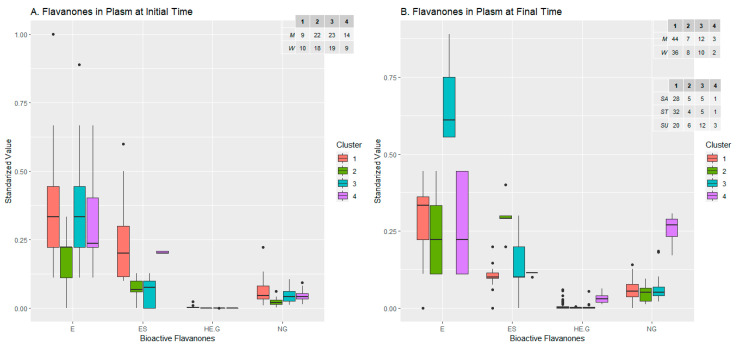
Levels of bioactive flavanones molecules within the groups generated by clustering analysis before (**A**) and after (**B**) beverage consumption, and composition of groups in terms of the sexes and sweeteners added to the drink.

Moving on to the final sampling time, four clusters were displayed again, but in this case they did not overlap as much as in the initial-time distribution (Figure 5B). There were three highly dispersed clusters (1, 2 and 3) with cluster 1 containing most of the population and a small regulation by E and ES (to a lesser degree) compounds. Cluster 2 seemed to conform around the compound ES influence, and cluster 3 was under both E and ES effects. Lastly, cluster 4 had a strange shape again, but this time the influence was only provided by compound E. The examination of levels of flavanones presented in the different clusters at the final sampling time (Figure 6B) endorsed the interpretation of the last biplot: cluster 1, with most of the population, was influenced mainly by flavanone E, and cluster 2 had its mean level of ES higher than any other cluster. In addition, cluster 3, which contained the most prominent difference in terms of sweetener composition with its amount of sucralose-sweetened beverages, was mostly influenced in its composition by flavanone E. This could reveal an interaction between the sucralose sweetener and E levels, already seen succinctly in the ANOVA analysis. Another insight that can be extracted is the relationship between men and E levels, by examining cluster 1, which had a male majority and a high level of compound E (again, as seen slightly in ANOVA). Taking a closer look at cluster 4 at the initial time and final time, only observations 18 and 61, which were females with different sweeteners, coincided, which did not give rise to a firm explanation for the strange shape of the clusters. The reason seems to lie in their common E-S value, fixed at a standardised value of 0.2 at the initial time and 0.1 at the final time. 

## 3. Discussion

The present study takes over from previous works [3,7,8], whose results revealed a high presence of bioavailable polyphenols in the beverage studied, and the influence of alternative sweeteners to sucrose on their bioavailability. Therefore, the work measured the interaction of the sex factor in the study with the consumption of the beverage and the added sweetener, using applied-statistics tools to extend the previous results. 

From the initial pool of compounds, some metabolites could be identified as elements in which concentration was influenced by sex and by the interaction between sex and sweetener. Regarding the anthocyanin family, these metabolites would be mainly positively influenced in men who ingested the sucrose-sweetened beverage, with total CA, DHPAA-GG, DHPAA-GS, DHPAA-SS and VA-GG being the most strongly concentration-increased metabolites. However, the strong regulation of stevia in women on total TFA reflected in the pairwise *t*-Test, an analysis that also yielded regulations on TFA-G and TFA-S of stevia and stevia/women, was striking. In the case of the flavanone data, the regulation of the beverage on E and N-G occurs especially in females, i.e., women who consumed the beverage saw increased concentrations of these metabolites at the final time, with respect to the initial time.

Sex differences in the regulation of phenolic compounds such as anthocyanins and flavanones have been determined in only a few studies [9,10]. Thus, it is difficult to compare the results obtained with the literature, since most studies consider and/or work only with male individuals. 

The effects of TFA in women have been studied in breast cancer [11], but there is not much other evidence, except for a study of the impact of TFA as an anti-obesity agent [12], which only slightly addresses sex differentiation; the effects of flavanone N-G have not been as well studied, unlike other flavanones [13]. However, N-G has been classified as an active phyto(xeno)estrogen [14], which could be the reason for its differential level observed in plasma. Looking at the sweeteners, the strongest relations arise in men, with sucrose and anthocyanin metabolites (CA, VA-GG, DPHAA, DPHAA-G). The relation sucrose-anthocyanin has been studied as a delay in the excretion of anthocyanin, due to the sucrose [15], which could explain the high levels in plasma. 

The results of the clustering at the initial time allowed us to determine a certain difference between the baseline levels of metabolites according to sex, prior to the consumption of the beverage, as the highest regulation of anthocyanins was observed in men. Once the data belonging to the moment when the drink has been consumed were analysed, the obtained results gave an insight into possible relationships between trial factors (sweetener, sex and time) and different concentrations of compounds, which could lead to deeper studies to reveal mechanisms of regulation associated with the concrete combinations of factors. For example, the possible relation between stevia and the levels of certain flavanones (E, N-G) in women showed one of these potential, but still hidden, mechanisms of regulation. In anthocyanins, a relationship between VA derivatives and DHPAA derivatives was observed in men. Regarding flavanones, it was possible to determine relationships between sucralose and compound E, and, independently, between the male sex and compound E itself, which could be another interfactorial hidden interaction.

As in any data-oriented study, pre-processing usually leaves out information from data. In addition to the main scope of the study (to examine if there are any difference between the sexes in the analysed drink metabolisation), a more detailed and accurate analysis is provided. Since certain metabolic compounds were not found in all the volunteers, gaps were generated, and various techniques were employed to deal with them, sometimes assuming a loss of information. 

To summarise, it would be interesting to study the mechanism of the metabolism of these compounds in both women and men with interactions with several sweeteners, and seek the reason for those differences. To obtain a bigger picture of the problem, a new perspective could be to analyse the cases of transgender people receiving hormonal treatment, since they have a different hormonal load and hormones could play an important role in (poly)phenolic metabolism.

As mentioned before, is not very common to use sex as a factor to describe metabolism, but the present work tries to take that approach. Lastly, the study of the interaction between sex and sweetener can give us even more insight into the differential processing in the metabolism of flavanones and anthocyanins, which would make us understand better how the bioavailability, bioaccessibility, and bioactivity of those compounds work.

## 4. Materials and Methods

### 4.1. Experimental Phase

The volunteers were provided with fresh drinks for their intake every day for two months. At the start and end of the experiment, urine and blood samples were collected to assess the levels of (poly)phenolic metabolites. The identification and quantification of these metabolites were achieved by UHPLC-ESI-QqQ-MS/MS [3].

### 4.2. Computational Phase

#### 4.2.1. Dataset

The dataset analysed included concentration of flavanones and anthocyanins metabolites present in the plasma samples, time of plasma collection, sex of the volunteers and sweetener added to the beverages. From this point, the factors considered, including some of the possible interactions with each other, were as follows: time (mere consumption of the beverage, independently of the other factors), sex–time (how the beverage affects metabolism by sex), sweetener–time (differential effect of sweetener on metabolism), and sex–sweetener–time (how drinking a beverage with an added sweetener affects the metabolism of a particular sex).

#### 4.2.2. Pre-processing

All considered variables were normalised (scaled from zero to one) to perform the analysis, correcting magnitude differences among them and performing a fair comparison of their possible effects or responses. Descriptive statistics (e.g., mean, standard deviation, quartiles, etc.) for all the considered variables as different tests were performed, to evaluate skewness, kurtosis, homoscedasticity, and normality of the data, to check the viability of the ANOVA technique. The obtained results are available in Supplementary Data (Table S3). 

#### 4.2.3. Analysis of Variance (ANOVA)

The analysis-of-variance technique measures the effect of one or more factors (with two or more levels) over the mean of a continuous variable. In this case, because of the nature of the data, the repeated-measures (also known as paired) ANOVA was applied. The three-way paired ANOVA was applied to assess the effects of the different involved factors (time, sweetener, and sex) and their possible interactions on different metabolic values. The only condition for performing this analysis is sphericity, which means that the variance of the difference between every pair of variables compared shall be equal. To assess this, Mauchly’s test was performed and, in case of violation of sphericity, the Greenhouse–Geisser correction was applied [16,17].

To check the veracity of the cases where a significant influence of the factors over the responses was found, and to look at the relationship between levels of factors even if no significance in ANOVA was found, post hoc paired *t*-tests were applied. In doing so, the mixed effects from the three factors were decomposed into two and one, and thus the significance of the difference between the means of the different groups was analysed, applying the Benjamini–Hochberg procedure to decrease the false discovery rate [18], as it has been proposed as the more efficient for biological data [19].

#### 4.2.4. Data Imputation

As discussed above, the empty gaps in data are an issue for many computational functions and algorithms [20]. To address this, data imputation was applied. It is a technique for replacing the missing data with a substitution, in order to retain most of the information of a dataset, widely applied in different fields [21,22]. For this task, the “simputation” R package [23] was used, more specifically its function to perform imputation through regression trees. The imputation was applied after the ANOVA analysis, since both the functions used to perform feature selection and clustering required data sets without gaps. In the case of ANOVA, the function applied belongs to the “rstatix” package [24], which is able to automatically remove, for every compound analysed, the individuals (the rows in terms of a table) with missing values. If the function leaves out more than 20 individuals, this is indicated in the results table.

#### 4.2.5. Feature Selection

Similar to dimensionality-reduction techniques, feature selection is a method for choosing variables, based on different types of importance/significance measures. It is used to remove redundant or non-significant variables from the modelling to improve the performance of multivariate techniques [25]. The algorithm chosen for this task was the Boruta algorithm for feature selection [26], which provides a robust and statically grounded feature-selection process. To simplify its procedure, the algorithm considered as input a formula in which a target variable was defined (sex for the initial sampling times, or sex and sweetener for the final sampling times, in this case), and the predictor variables to be selected or discarded. Later, it evaluated a model based on the random-forest algorithm, by adding one randomised variable for each predictor variable, determining whether each original variable performed better than its randomised counterpart. This process was repeated several times (thus avoiding possible failures associated with randomization), to generate a binomial distribution in which each variable could be accepted, discarded or left uncertain. The accepted variables became part of the following clustering model. 

#### 4.2.6. Clustering

The clustering technique, which belongs to the unsupervised learning from machine learning [27], allows us to find patterns and define groups of interest, based on an arithmetic distance (in this study the Manhattan distance for all cases was the selected, since it is the most suitable for data with many outliers [28]) between the standardised values of the variables considered for the volunteers that have consumed the beverage. Through these, associations between compounds were obtained at different times, and it was possible to observe how individuals were grouped throughout the study. Clustering has been widely used in food technology [29,30,31]. Several clustering methods can be applied, depending on the shape of the data and the scope of the study. To fix the most accurate number of groups (so-called clusters) the R package “NbClust” [32] was applied, while the benchmark “clValid” from the homonym R package [33] was used to assess different methods and choose the one that better fit our data. The number of clusters and the clustering method were evaluated for each sampling time in each dataset, obtaining four different methods: k-means, PAM (belonging to the non-hierarchical clustering), DIANA (belonging to the hierarchical clustering) and model-based clustering [34]. The choices made in terms of technique and number of clusters are listed in Table 3.

The results for every method can be represented in a biplot, a particular chart consisting of two elements: the first is a PCA projection, a representation that first aggregates all variables into other variables called principal components, which allows them to be represented in two dimensions, thus enabling the study of the distribution of the groups in the principal components that group most of the variability of the variables in the data set. The other element is a representation of every variable as an arrow, whose direction represents its contribution to the clusters composition and whose longitude indicates the amount of such contribution to the clusters it affects. A biplot chart can be a very visual means of understanding the results obtained, allowing one to visualise how the variables contribute to a given arrangement of individuals, based on clustering analysis, and thus to evaluate the meaning of the generated groupings, and thus to look for patterns and associations between variables (compounds, in this case).

## 5. Conclusions

The influence of the sex factor on the bioavailability of (poly)phenols has been analysed through a longitudinal intervention-study involving the ingestion of a beverage composed of citrus fruits (with a high presence of flavanones) and maqui (with a high presence of anthocyanins), and sweetened with sucrose, sucralose or stevia.

In summary, two major points were observed: the differences between both sexes, and their relationship with diverse sweeteners. It is possible to describe the interaction of several compounds differently according to sex, which could lead to new studies to observe those interactions, presumably based on hormone or physiological regulation. 

Regarding the issue proposed, the differences between men and women, independently of the sweetener, were evidenced, such as in the TFA and N-G for women or DHPAA for men (which is not influenced by sucrose, like many other anthocyanins).In addition, there were differences regarding the sweetener; for example, the CA and VA-GG in men with sucrose. The intra-relation between the metabolites analysed is not clear, but is approaching a precise definition, as seen in the case of DHPAA and VA derivatives. In addition, some of the insights discovered by the clustering approach could be translated into new studies, to analyse the potential ways of internal regulation, such as the stevia regulation over N-G or the potential link between stevia, women, and the levels of VA-GG, CA, and DHPAA-G. 

## Figures and Tables

**Table 1:** *p*-value for the ANOVA and pairwise t-test analysis of plasma-anthocyanin results. In bold, those *p*-values < 0.05 from ANOVA. In the *t*-test results, (+) means a positive relation. More than one symbol indicates a higher grade of relation, based on a *p*-value < 0.01 for (++) and *p*-value < 0.001 for (+++). CA-sulfate was excluded for not showing significant results, either in ANOVA or in the *t*-test. Abbreviations: M, male; F, female; SA, sucrose; SU, sucralose; ST, stevia.

Compounds	Factors and Interactions (*p*-Value)			
	Time	Sex–Time	Sweetener–Time	Pairwise t-Test
CA	**1.68 × 10^−10^**	5.87 × 10^−1^	1.32e × 10^−1^	M/SA(+++), M/SU(++), F/SA(++), F/ST(++), M(+++), F(+++), SA(+++), ST(++), SU(++). T(+++)
CA-G	**3.20 × 10^−2^**	**3.7 × 10^−2^**	7.02 × 10^−1^	M/SU(+), M(+), T(+)
Total CA	**4.47** ** × 10^−8^**	6.70 × 10^−2^	3.45 × 10^−1^	M/SA(+++), M/SU(++), F/ST(+), M(+++), F(+++), T(+++)
DHPAA	**1.55** ** × 10^−7^**	**8.00** ** × 10^−3^**	8.66 × 10^−1^	M/SA(+++), M/ST(++),M/SU(+++), M(+++), F(+), SA(++), ST(++). SU(+++), T(+++)
DPHAA-G	**5.28** ** × 10^−7^**	6.50 × 10^−2^	**1.45** ** × 10^−4^**	M/SA(+++), M/SU(++), M(+++), F(+), SA(+++), SU(++)
DHPAA-GG	**6.52** ** × 10^−6^**	3.39 × 10^−1^	**2.87** ** × 10^−5^**	M/SA(+++), F/SA(+), F/ST(+), M(+++), F(+), SA(+++), ST(+), T(+++)
DPHAA-GS	**4.19** ** × 10^−5^**	**5.00** ** × 10^−3^**	**1.00** ** × 10^−3^**	M/SA(++), M/SU(+), M(+++), SA(+++), T(+++)
DHPAA-SS *	1.25 × 10^−1^	9.8 × 10^−2^	5.17 × 10^−1^	M/SA(++), M(+)
Total DHPAA	**9.07** ** × 10^−13^**	**4.00** ** × 10^−6^**	8.20 × 10^−2^	M/SA(+++), M/ST(+++), M/SU(+++), F/SA(++), F/ST(++), M(+++), F(+++), SA(+++), ST(+++), SU(+++), T(+++)
TFA-G *	1.09 × 10^−1^	8.59 × 10^−1^	7.74 × 10^−1^	ST(+)
TFA-S	9.7 × 10^−2^	4.38 × 10^−1^	8.09 × 10^−1^	F/SA(++), F/ST(++), ST(+)
Total TFA	**5.00 × 10^−2^**	3.08 × 10^−1^	8.33 × 10^−1^	F/ST(+++), F(+), ST(++), T(+)
VA-GG	**6.64 × 10^−15^**	8.35 × 10^−1^	**1.17 × 10^−10^**	M/SA(+++), M/SU(++), F/SA(++), F/SU(+), M(+++), F(+++), SA(+++), SU(++), T(+++)
VA-S *	**2.47** ** × 10^−4^**	5.95 × 10^−1^	**5.00** ** × 10^−3^**	F/SA(+), F/ST(+), M(+), SA(+), ST(+), T(++)
VA-GS	**7.97** ** × 10^−4^**	2.78 × 10^−1^	1.15 × 10^−1^	M/SA(+), F/SA(+), M(++), SA(++), T(+++)
VA-SS	**5.00 × 10^−3^**	6.29 × 10^−1^	4.48 × 10^−1^	ST(+,), T(+)

* More than 20 individuals were removed by the algorithm for containing missing values.

**Table 2:** *p*-value for the ANOVA and pairwise t-test analysis of flavanones results. In bold, those *p*-values < 0.05 from ANOVA. In the *t*-test results, (+) means a positive relation. More than one symbol indicates a higher grade of relation, based on a *p*-value < 0.01 for (++) and *p*-value < 0.001 for (+++). Abbreviations: M, male; F, female; SU, sucrose, ST, stevia; NR, non-relevant.

Flavanones	Factors and Interactions (*p*-Value)			
	Time	Sex–Time	Sweetener–Time	Pairwise *t*-Test
E	**9.00 × 10^−3^**	6.78 × 10^−1^	5.63 × 10^−1^	M(+), SU(+), T(++)
E-sulfate *	4.14 × 10^−1^	3.03 × 10^−1^	3.74 × 10^−1^	NR
HE-G	1.15 × 10^−1^	3.40 × 10^−1^	5.94 × 10^−1^	M/ST(+), F/ST(+), M/SU(+), F(++), ST(++), SU(++)
N-G	**1.8** ** × 10^−4^**	3.51 × 10^−1^	6.73 × 10^−1^	F/SA(+), M/ST(+++), M/SU(+), F/SU(+), M(++), F(+++), ST(+++), SU(+++), T(+++)

* More than 20 individuals were removed by the algorithm for containing missing values.

**Table 3:** Parameters associated with the clustering techniques applied to the datasets of the work, selected by ClValid benchmark.

Time–Dataset	Clustering Technique	Number of Clusters
Initial–Flavanones	Model-Based	4
Final–Flavanones	DIANA	4
Initial–Anthocyanin	PAM	6
Final–Anthocyanin	K-means	6

**Table 4:** Anthocyanin selected by Boruta algorithm.

Sample Time	Anthocyanin Selected
Initial	Total CA, TFA-S, Total TFA, VA-SS, Total VA
Final	DHPAA-GS, TFA-S, Total TFA, VA-GG, VA-GS

## Data Availability

Data sharing is not applicable to this article.

## Supplementary Materials

The following supporting information can be downloaded at https://www.mdpi.com/article/10.3390/ijms24032140/s1, Table S1: Pairwise *t*-test results from anthocyanins; Table S2: Pairwise *t*-test results from flavanones; Table S3: Descriptive statistics; Figure S1: Full of compounds boxplot from anthocyanins.
