# Supplementary material for: Bioactive Compounds in Plasma as a Function of Sex and Sweetener Resulting from a Maqui-Lemon Beverage Consumption Using Statistical and Machine Learning Techniques
Source: Int J Mol Sci. 2023 Jan 21;24(3):2140. doi: 10.3390/ijms24032140 (PMC9917032; doi:10.3390/ijms24032140)
Supplement: Supplementary file 1 [file ijms-24-02140-s001.zip › ijms-2094841-supplementary.pdf]

Supplementary material articlePlasm

Table S1. Pairwise T-test results from anthocyanins

		Sweetener			
	**caffeic acid**	SA	ST	SU	1-way sex
Sex	M	0.000234		0.006	0.00000299
	F	0.002	0.003		0.00000932
	1-way sweetener	0.00000158	0.002	0.002	1.38e-10

		Sweetener			
	**CA-Gluc**	SA	ST	SU	1-way sex
Sex	M			0.046	0.01
	F				
	1-way sweetener				0.016

		Sweetener			
	**TOTAL CA**	SA	ST	SU	1-way sex
Sex	M	0.000473		0.002	0.00000825
	F		0.018		0.000339
	1-way sweetener				0.0000000189

		Sweetener			
	**DHPAA**	SA	ST	SU	1-way sex
Sex	M	0.003	0.001	0.00035	0.00000000911
	F				0.038
	1-way sweetener	0.005	0.001	0.000368	0.0000000217

		Sweetener			
	**DHPAA-Gluc**	SA	ST	SU	1-way sex
Sex	M	1.25e-4		3 e-3	0.0000185
	F				0.048
	1-way sweetener	0.000133		0.003	

		Sweetener			
	**DHPAA-di-Gluc**	SA	ST	SU	1-way sex
Sex	M	0.00041			0.000291
	F	0.02	0.029		0.016
	1-way sweetener	0.0000201	0.016		0.0000124

	DHPAA.Gluc.Sulfate	Sweetener SA	ST	SU	1-way sex
Sex	M	0.002		0.011	3.36e-5
	F				
	1-way sweetener	6.64e-4			0.000375

	DHPAA-di-Sulfate	Sweetener SA	ST	SU	1-way sex
Sex	M	0.007			0.021
	F				
	1-way sweetener				

	TOTAL DHPAA	Sweetener SA	ST	SU	1-way sex
Sex	M	3.31e-5	5.17e-4	1.04e-4	4.88e-11
	F	2.3 e-2	3.6 e-2		6.57e- 4
	1-way sweetener	0.00000288	0.0000568	0.000123	5.26e-13

	TFA-Gluc	Sweetener SA	ST	SU	1-way sex
Sex	M				
	F				
	1-way sweetener		0.017		

	TFA-Sulfate	Sweetener SA	ST	SU	1-way sex
Sex	M				
	F	0.004	0.003		
	1-way sweetener		0.02		

	TOTAL TFA	Sweetener SA	ST	SU	1-way sex
Sex	M				
	F		0.000704		0.02
	1-way sweetener		0.004		0.031

		Sweetener			
	**VA-GG**	SA	ST	SU	1-way sex
Sex	M	6.32e-7		5 e-3	0.000000147
	F	2.34e-4		1.8 e-2	0.0000363
	1-way sweetener	5.93e-10		2.37e- 4	2.9e-11

		Sweetener			
	**VA-Sulfate**	SA	ST	SU	1-way sex
Sex	M				0.028
	F	0.025	0.027		
	1-way sweetener	0.012	0.004		0.004

		Sweetener			
	**VA-Gluc-Sulfate**	SA	ST	SU	1-way sex
Sex	M	0.024			0.002
	F	0.021			
	1-way sweetener	0.001			0.000444

		Sweetener			
	**VA-di-Sulfate**	SA	ST	SU	1-way sex
Sex	M				
	F				
	1-way sweetener		0.033		0.017

Figure s1. Full of compounds boxplot from anthocyanins

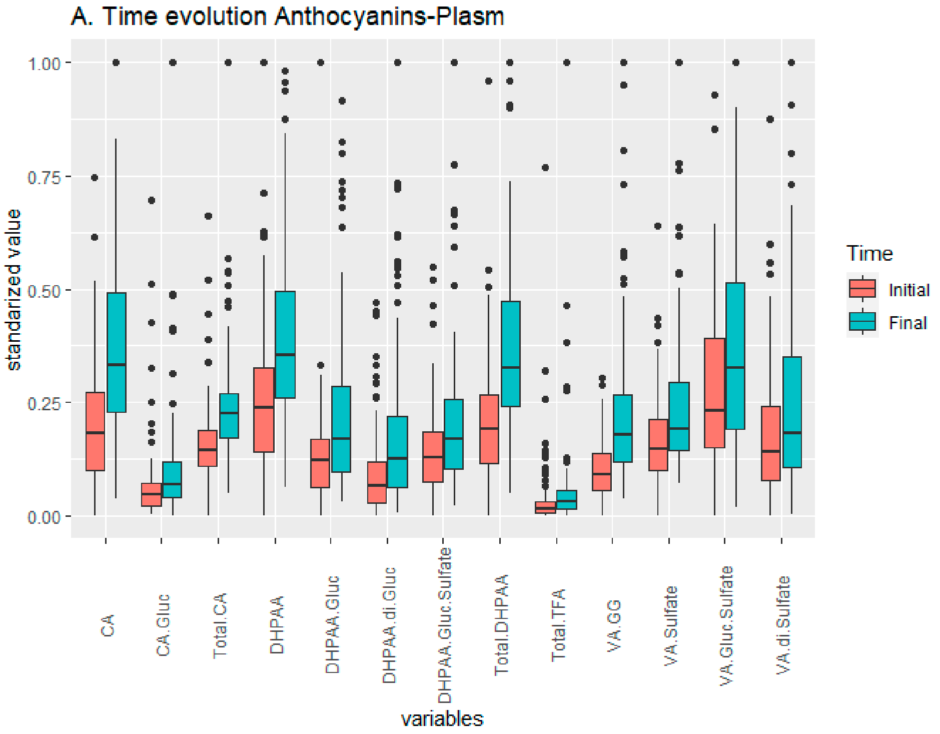

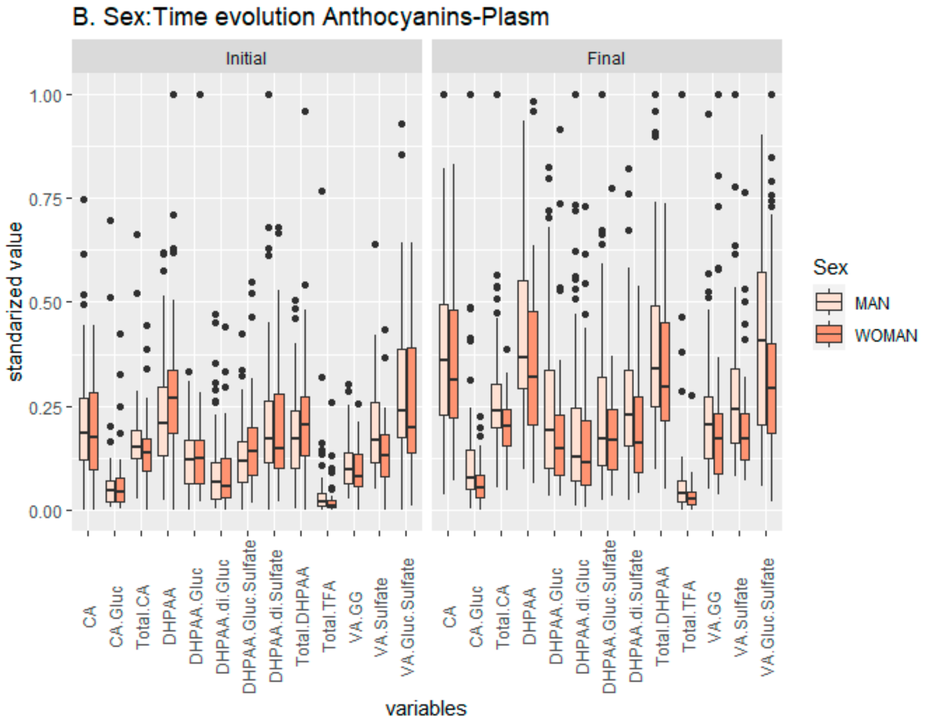

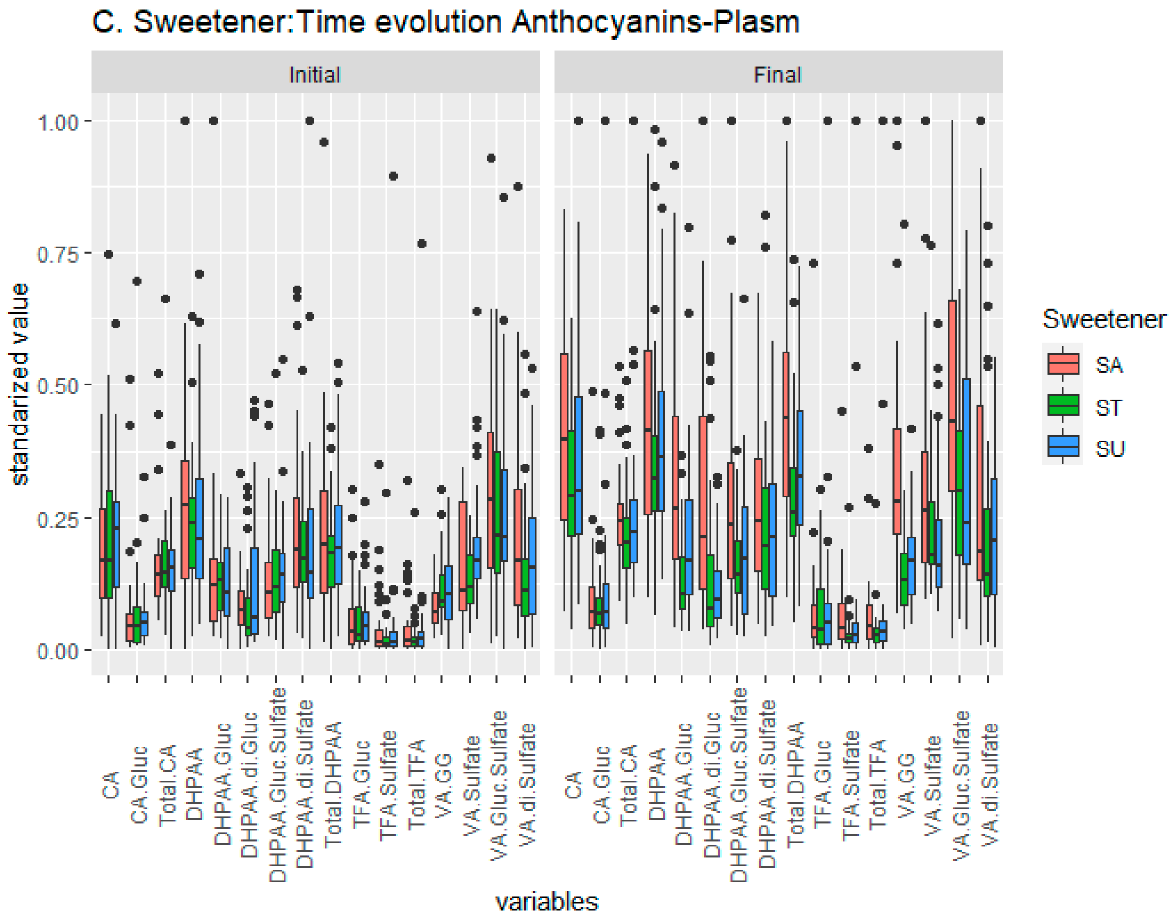

Table S2. Pairwise T-test results from flavanones

		Sweetener			
	**Eriodyctiol**	SA	ST	SU	1-way sex
Sex	M				0.017
	F				
	1-way sweetener			0.026	0.006

		Sweetener			
	**ES**	SA	ST	SU	1-way sex
Sex	M				
	F				
	1-way sweetener				

	Sweetener			
**HE-G**	SA	ST	SU	1-way sex

		SA	ST	SU	1-way sex
Sex	M		0.039	0.018	
	F		0.033		0.003
	1-way sweetener		0.004	0.003	

		Sweetener			
	**NG**	SA	ST	SU	1-way sex
Sex	M		0.0000592	0.013	0.006
	F	0.021		0.016	0.000621
	1-way sweetener		0.000641	0.000418	0.000109

Table S3. Descriptive statistics

Anthocyanins

	Minimum	Q1	Mean	Trimmed Mean	Median	Mode	Variance	Desviation	Q3	Maximum	Symmetry	Kurtosis
CA	0	0.14457831	0.28070238	0.27378894	0.24096386	0.22891566	0.0326007	0.18055663	0.38554217	1	0.97259014	0.86744927
CA.Gluc	0	0.025	0.08263513	0.07147674	0.05535714	0.07142857	0.01165026	0.10793636	0.09375	1	4.36276121	26.3121707
CA.Sulfate	0	0.15370184	0.28104441	0.27127795	0.22580645	0.17741935	0.03420493	0.18494575	0.37096774	1	1.39587125	1.89716173
Total.CA	0	0.13279133	0.19927441	0.19235451	0.17750678	0.14363144	0.01235968	0.11117408	0.24119241	1	2.46557979	11.745235
DHPAA	0	0.18960938	0.32231099	0.31415254	0.2896875	0.0475	0.03625776	0.19041471	0.40835938	1	1.12044748	1.42301019
DHPAA.Gluc	0	0.07746849	0.17732986	0.164724	0.13760504	0.11892583	0.02449436	0.15650675	0.21691176	1	2.4442986	7.43579184
DHPAA.di.Gluc	0	0.04471154	0.13726093	0.12535993	0.08205128	0.07226583	0.0227138	0.150711	0.15721154	1	2.39808473	6.88287578
DHPAA.Gluc.Sulfate	0	0.09375	0.17428358	0.16428304	0.14285714	0.1388866	0.01759301	0.13263865	0.21173469	1	2.36936706	8.5364687
DHPAA.di.Sulfate	0	0.11809045	0.22519777	0.21602806	0.19346734	NA	0.0261647	0.16175506	0.28831658	1	1.6254623	3.51954486
Total.DHPAA	0	0.15711948	0.28547576	0.2765789	0.24514847	0.12859481	0.03482791	0.18662236	0.36591069	1	1.26405188	1.95720035
TFA.Gluc	0	0.01482702	0.06944989	0.05947039	0.03953871	NA	0.0112219	0.10593347	0.08841296	1	5.19455133	37.2889477
TFA.Sulfate	0	0.00952212	0.04479858	0.03214121	0.02008458	0.02008458	0.01039249	0.10194358	0.03993672	1	6.58927616	51.3038933
Total.TFA	0	0.01024132	0.04553054	0.03420836	0.0228666	0	0.00898606	0.09479484	0.04499124	1	6.69835957	54.9302245
VA	0	0.12150127	0.24588932	0.23694194	0.19508058	NA	0.03315775	0.18209271	0.34033079	1	1.39934808	2.95880962
VA.GG	0	0.07760652	0.16266258	0.15077732	0.12492637	0.09670135	0.01942104	0.13935939	0.2080429	1	2.81869847	11.124469
VA.Sulfate	0	0.11691023	0.21337714	0.20418332	0.17397356	NA	0.02134362	0.14609455	0.26026444	1	2.06580918	5.94637362
VA.Gluc.Sulfate	0	0.1696325	0.32320899	0.31623582	0.2762089	0.14390716	0.04091964	0.20228603	0.4459381	1	0.87168194	0.25233397
VA.di.Sulfate	0	0.09215362	0.2109309	0.19946066	0.15123117	0.09735916	0.03311887	0.1819859	0.28574054	1	1.65999669	3.05267265

Total.VA	0	0.1076294	0.20260815	0.19227331	0.17057139	0.09601284	0.02230184	0.14933801	0.24557465	1	2.2016654	7.02068395

Flavanones:

	Minimum	Q1	Mean	Trimmed Mean	Median	Mode	Variance	Desviation	Q3	Maximum	Symmetry	Kurtosis
E	0	0.22222222	0.32407075	0.31653763	0.33333333	0.22222222	0.03352035	0.18308564	0.44444444	1	0.94455012	0.94310987
ES	0	0.076	0.12492907	0.11729835	0.1	0.1	0.01219346	0.11042401	0.2	1	2.78849893	16.1958255
Total.E	0	0.125	0.35119048	0.34375	0.25	NA	0.07716318	0.27778262	0.59375	1	0.65083916	-0.62919089
HE.G	0	0.000199	0.00761996	0.00260899	0.00059701	0.000199	0.00409201	0.06396884	0.00199005	1	15.0642182	230.56131
NG	0	0.02634979	0.0569768	0.04988577	0.043483	0.01360692	0.00546371	0.07391692	0.06529456	1	8.88956198	105.385564
